# A Tetravalent Recombinant Subunit Vaccine Provides Protection Against Mixed Challenges with Four *Eimeria* Species in Chickens

**DOI:** 10.3390/ani16071087

**Published:** 2026-04-01

**Authors:** Xiao Ma, Xichen Zhang, Jianhua Li, Pengtao Gong, Xiaocen Wang, Xin Li, Xu Zhang, Tao Zhang, Shuqin Cheng, Nan Zhang

**Affiliations:** 1 State Key Laboratory for Diagnosis and Treatment of Severe Zoonotic Infectious Diseases, College of Veterinary Medicine, Jilin University, Changchun 130062, China; 2 Department of Agriculture and Animal Husbandry, Handan Vocational College of Science and Technology, Handan 056046, China

**Keywords:** *Eimeria* mixed infections, *Pichia pastoris*, subunit vaccine, immune protection, safety

## Abstract

Chicken coccidiosis is a parasitic disease caused mainly by *Eimeria tenella*, *Eimeria acervulina*, *Eimeria maxima*, and *Eimeria necatrix*, with most cases presenting as mixed infections. Currently, although a subunit vaccine (CoxAbic) targeting *Eimeria maxima* via maternal immunization is commercially available, no genetically engineered multivalent subunit vaccine exists against mixed infections caused by these four *Eimeria* species simultaneously. Therefore, we developed a tetravalent recombinant subunit vaccine (designated TEIN) by fusing key antigen genes (TA4, 3-1E, IMP1, NA4) from these four *Eimeria* species and expressing the construct in *Pichia pastoris*. A total of 500 chickens were randomly allocated into 25 experimental subgroups (*n* = 20 each), consisting of five groups (control, challenged, adjuvant, pPIC9K, and TEIN) and five challenge conditions (infection with *Eimeria tenella*, *Eimeria acervulina*, *Eimeria maxima*, *Eimeria necatrix*, or a mixture of four species). Immunization was performed via leg intramuscular injection at 14 and 21 days of age. At 28 days of age, all chickens except the controls were orally challenged with 1 × 10^4^ sporulated oocysts. Statistical analysis was performed using one-way or two-way ANOVA as appropriate. Results showed that chickens vaccinated with the TEIN subunit vaccine exhibited significantly elevated serum levels of IgY, IL-2, IL-10, and IFN-γ, as well as an increased splenic lymphocyte CD4^+^/CD8^+^ ratio. The anticoccidial indices (ACI) against *Eimeria tenella*, *Eimeria acervulina*, *Eimeria maxima*, and *Eimeria necatrix*, and their mixed infection reached 174.82, 174.58, 174.41, 180.61, and 175.95, respectively. Moreover, no significant differences were observed in hematological parameters, serum biochemical markers, or histopathological findings between the vaccinated and control groups. These results demonstrate the vaccine’s potential as a promising candidate for controlling mixed coccidial infections.

## Simple Summary

Chicken coccidiosis is a widespread parasitic disease that causes major economic losses in poultry farming. In the field, chickens are often infected by several types of *Eimeria* parasites at the same time, but currently available commercial vaccines are mainly live oocyst-based products, which carry potential safety risks including reversion to virulence and vaccine strain transmission. To tackle this problem, we developed a subunit vaccine called TEIN, which combines key proteins from four common *Eimeria* species. Using molecular tools, recombinant proteins were produced, and the vaccine efficacy was tested under controlled experimental conditions. Vaccination significantly elevated serum levels of cytokines and increased the splenic lymphocyte CD4^+^/CD8^+^ ratio. In homologous challenge experiments using each of the four *Eimeria* species individually or in combination, vaccinated chickens showed better weight gain, lower parasite counts, and fewer intestinal lesions, demonstrating that the vaccine conferred strong protection. These results demonstrate that the TEIN vaccine provides effective protection against both single and mixed *Eimeria* infections under laboratory conditions, but its efficacy under field conditions requires further investigation.

## 1. Introduction

Chicken coccidiosis is a protozoan parasitic disease caused by *Eimeria* species infecting the intestinal tract of chickens. It is one of the most widespread and economically devastating diseases in poultry production, causing annual global losses exceeding £10 billion and underscoring the urgent need for effective control strategies [1,2]. The control of chicken coccidiosis mainly relies on anticoccidial drugs and live vaccines [3,4]. However, the long-term use of anticoccidial drugs not only leads to drug residues, which threaten food safety, but also may lead to drug resistance in chicken coccidia, increasing the difficulty of preventing and controlling chicken coccidiosis [5,6]. Although live vaccines offer some protective benefits, their application is limited by risks such as reversion to virulence and environmental spread of vaccine strains [7,8,9]. Beyond these concerns, the production of live *Eimeria* vaccines requires the use of live birds to propagate the parasites, which raises ethical concerns and increases manufacturing costs [10]. Additionally, these vaccines have a relatively short shelf life due to the limited viability of oocysts during storage [11]. Genetically engineered vaccines, such as DNA vaccines and subunit vaccines, offer several advantages over conventional live vaccines, including well-defined antigenic components, the absence of live pathogens and the potential for cost-effective large-scale production [12,13]. Therefore, a safe and efficient genetically engineered vaccine needs to be developed for the prevention and control of chicken coccidiosis.

Genetically engineered vaccines for chicken coccidia mainly include subunit vaccines, DNA vaccines, and live vector vaccines [14,15,16]. Among them, subunit vaccines have become a critical strategy for controlling chicken coccidiosis because of their absence of live pathogens and high stability [17,18]. Currently, the only commercially available subunit vaccine for routine use in coccidiosis control is CoxAbic. This vaccine is based on affinity-purified gametocyte antigens from *Eimeria maxima* (*E. maxima*) and is administered to breeding hens to provide passive immunity to their offspring via maternal antibody transfer [19]. In recent years, significant progress has been made in the development of recombinant subunit vaccines and recombinant multivalent subunit vaccines, with research efforts primarily focused on the screening and optimization of antigen targets, multi-antigen combination strategies [20,21]. Recombinant fusion proteins containing multiple antigens tend to induce broader protective immunity than those with a single antigen, offering a promising approach to overcome the limitations of single-antigen formulations [22]. For instance, a recent study reported that the elongation factor 2 (EF2) antigen provided good protection against individual *Eimeria* species but showed poor efficacy against mixed infection [23]. Another study demonstrated that a combination of three gametocyte antigens (rEnGAM22, rEnGAM56-T, and rEnGAM59) induced stronger immune responses and higher protective efficacy than any single antigen alone [24]. Given the advances in subunit vaccines, the selection of an appropriate expression system is important to achieve the efficient expression of exogenous proteins [25]. The main vector systems commonly used for the expression of chicken coccidia antigenic proteins are *Escherichia coli*, *Bacillus subtilis*, and yeast-based platforms [26,27,28]. Among them, *Pichia pastoris* (*P. pastoris*) has been used for a long time to produce recombinant proteins because of its rapid fermentation kinetics and low cost [29,30]. The *P. pastoris* expression system can perform efficient secretory expression of exogenous proteins, and its glycosylation is more suitable for use with mammalian cells, which is conducive to improving the immunogenicity of proteins, thereby establishing it as an ideal platform for developing subunit vaccines [31,32].

Chicken coccidiosis is caused mainly by mixed infections of *Eimeria tenella* (*E. tenella*), *Eimeria acervulina* (*E. acervulina*), *E. maxima*, and *Eimeria necatrix* (*E. necatrix*). Since most cases involve co-infections, genetically engineered vaccines targeting only a single *Eimeria* species are inadequate for effective coccidiosis control [33]. In response, studies have shown that through the recombinant expression of protective genes of different coccidia, the constructed multivalent vaccine can provide immune protection against multiple coccidial infections and effectively prevent mixed infection with multiple coccidia [34]. This multivalent vaccine-based strategy can overcome the limitations of monovalent vaccines and improve the protective efficacy of vaccines against multiple coccidial infections [35].

The TA4 gene of *E. tenella*, the 3-1E gene of *E. acervulina*, the IMP1 gene of *E. maxima,* and the NA4 gene of *E. necatrix* are used as candidate antigens, which can provide partial protection against infections by *E. tenella*, *E. acervulina*, *E. maxima*, and *E. necatrix*, respectively. However, most existing studies have focused on evaluating the protective efficacy of these antigens individually. There is a lack of research on the tandem expression of these four specific protective antigens (TA4, 3-1E, IMP1, NA4) in a single construct, particularly using the *P. pastoris* expression system, which offers better post-translational modification capabilities. Furthermore, whether such a tandemly expressed multi-antigen construct can induce effective immune responses against concurrent infections with all four *Eimeria* species has not been investigated, leaving a significant gap in developing effective multivalent subunit vaccines for mixed coccidial infections. This TEIN vaccine was evaluated for its immunoprotective efficacy and safety in chickens challenged with four *Eimeria* species, aiming to provide a potential candidate vaccine for mixed *Eimeria* infections.

## 2. Materials and Methods

### 2.1. Parasite Strains, Vectors, and Experimental Animals

Sporulated oocysts of *E. tenella* were maintained in our laboratory, while those of *E. necatrix* and *E. acervulina* were gifted by Prof. Jianping Tao (Yangzhou University, China), and the sporulated oocysts of *E. maxima* were donated by Prof. Xiangrui Li (Nanjing Agricultural University, China). All *Eimeria* strains were propagated via passage in coccidia-free chickens prior to the experiment to ensure viability, and freshly sporulated oocysts stored in 2.5% potassium dichromate at 4 °C for less than three months were used for all challenge infections. The pPIC9K plasmid was used as the expression vector for vaccine construction and was maintained in our laboratory. Newly hatched white broilers were maintained in sanitized coccidia-free isolation units throughout the study period. All animal enclosures and equipment were subjected to rigorous disinfection protocols. The birds were provided ad libitum access to anti-coccidial-free feed and water throughout the experimental period. At the beginning of the study, all chickens were randomly assigned to experimental groups. All animal experiments were conducted strictly following the Regulations for the Administration of Laboratory Animals and approved by the Experimental Animal Welfare Ethics Committee of Jilin University (Ethical Approval No. SY202202101).

### 2.2. Reagents and Kits

The following reagents were used: restriction enzymes EcoRI and NotI; rTaq DNA polymerase; dNTP mix; DNA Gel Extraction Kit (Takara, Shiga, Japan); Reverse Transcription Kit (TaKaRa, Shiga, Japan); HRP-conjugated 6×His-tag antibody; One-Step Rapid Cloning Kit (Yeasen, Shanghai, China); Plasmid Miniprep Kit (TransGen, Beijing, China); Yeast Genomic DNA Extraction Kit (Tiangen, Beijing, China); Lyticase (Tiangen, Beijing, China); BCA Protein Assay Kit (Thermo Fisher Scientific, Waltham, MA, USA); TMB Single-Component Substrate, ELISA Coating Buffer, and ELISA Stop Solution (Solarbio, Beijing, China); Chicken IL-2, IL-10 and IFN-γ ELISA kits (Fankew, Shanghai, China).

### 2.3. Preparation of the TEIN Subunit Vaccine

In this study, *P. pastoris* was chosen, as it performs post-translational modifications, such as glycosylation, and correct folding. It also secretes the protein, which simplifies downstream purification with lower production costs [36]. Flexible GGGGS linkers were inserted between the four antigens to minimize steric hindrance. The TA4 gene of *E. tenella* (M21004.1), the 3-1E gene of *E. acervulina* (AF113613.1), the IMP1 gene of *E. maxima* (KP642747.1), and the NA4 gene of *E. necatrix* (EU523548.1) were retrieved from NCBI and codon optimized for *P. pastoris*. These four genes were tandemly fused using GGGGS linkers (designated TEIN: TA4-3-1E-IMP1-NA4) and subsequently cloned and inserted into pPIC9K through seamless cloning (Figure S1). The recombinant plasmid pPIC9K-TEIN was linearized with SacI and transformed into *P. pastoris* GS115 (now reclassified as *Komagataella phaffii*) competent cells. Primary transformants were selected on histidine-deficient minimal dextrose medium, and high-copy number recombinant strains were obtained through gradient G418 selection [37]. Positive clones were verified by conducting polymerase chain reaction (PCR) and cultured in yeast extract peptone dextrose (YPD) medium. The cells were grown in buffered minimal glycerol medium with yeast extract (BMGY) at 30 °C with shaking (250 rpm), followed by centrifugation for harvesting. The cell pellet was resuspended in buffered methanol-complex medium with yeast extract (BMMY) to an optical density at 600 nm (OD600) of 1.0 and induced with 1% methanol (maintained by daily supplementation) for 120 h. After induction, the culture was centrifuged at 4 °C and 8000 rpm for 15 min to collect the supernatant. The supernatant was filtered through a 0.22 μm sterile filter and purified via Ni-NTA affinity chromatography. The eluted protein was buffer-exchanged and concentrated via ultrafiltration (30 kDa MWCO) to remove imidazole, followed by endotoxin removal using an endotoxin removal kit (Biorab, Beijing, China) according to the manufacturer’s instructions. The protein concentration was then determined using the bicinchoninic acid (BCA) method, and the purified TEIN protein was reconstituted with an equal volume of aluminum hydroxide adjuvant (Solarbio, Beijing, China) at 400 μg/mL for immunization.

### 2.4. Evaluation of Protection Against Challenge

To assess the protective efficacy of the TEIN subunit vaccine against single or mixed infections with *E. tenella*, *E. acervulina*, *E. maxima*, and *E. necatrix*, one-day-old chickens were randomly allocated into five main groups. The study included five treatment groups (control, challenged, adjuvant, pPIC9K, and TEIN) and five challenge conditions (four single-species infections and one mixed infection), resulting in a total of 25 experimental subgroups with 20 chickens per subgroup (500 birds in total). This group size was selected to ensure sufficient statistical power for evaluating protective efficacy indicators following challenge (Table 1). The chickens in the control and challenged groups received no treatment at 14 and 21 days of age, whereas those in the adjuvant group, pPIC9K group, and TEIN group were intramuscularly injected into the leg muscle with aluminum hydroxide adjuvant, pPIC9K empty vector protein, or TEIN subunit vaccine, respectively, at these time points. All groups except the control group were challenged with single or mixed *Eimeria* species at 28 days of age. The immunoprotective efficacy of the TEIN subunit vaccine was evaluated by measuring serum anti-TEIN IgY levels, serum cytokines, the CD4^+^/CD8^+^ ratio in the spleen, and the anticoccidial index in each group. To minimize bias, all outcome assessments were performed by personnel blinded to the group assignments.

#### 2.4.1. Serum Anti-TEIN IgY Antibody Quantification

Whole blood samples were collected from the wing vein of chickens in each group at 28 days of age using sterile syringes, following the method described by Neepa [38]. Five chickens were randomly selected from each subgroup for antibody analysis. After the samples were left undisturbed at room temperature for serum separation, the serum was collected by centrifugation at 3000 rpm (850× *g*) for 15 min. TEIN-specific IgY antibody levels were measured via indirect ELISA [39]. Briefly, 96-well plates were coated with TEIN protein (1 μg/well in ELISA coating buffer, pH 9.6, Solarbio, Beijing, China) and incubated overnight at 4 °C. Following three washes with PBST, the plates were blocked with 5% (*w*/*v*) skim milk in PBST at 37 °C for 2 h and washed again three times with PBST. Each serum sample was tested in triplicate wells. Test sera (1:100 dilution) were added and incubated at 37 °C for 2 h. After three additional PBST washes, 100 μL of horseradish peroxidase (HRP)-conjugated rabbit anti-chicken IgY secondary antibody (1:5000 dilution in PBST; Earthox, Burlingame, CA, USA) was added to each well and incubated at 37 °C for 1 h. The 1:5000 dilution was selected based on the manufacturer’s recommended working range for ELISA. Following five washes, 100 μL of TMB substrate solution (Solarbio, Beijing, China) was added to each well and incubated at 37 °C in the dark for 15 min. The reaction was stopped with ELISA Stop Solution (Solarbio, Beijing, China), and the OD450 was measured within 15 min using a microplate reader (DeTie, Nanjing, China). Antibody levels were expressed as OD450 values. To minimize inter-assay variation, all samples were analyzed on the same day using the same batch of reagents.

#### 2.4.2. Detection of Serum Cytokines

Whole blood samples were collected from chickens in each group when they were 21 and 28 days old. At each time point, three chickens were randomly selected from each subgroup for cytokine analysis. After serum separation at room temperature and centrifugation, serum samples were collected and analyzed for IL-2, IL-10, and IFN-γ levels using commercial ELISA kits (Fankew, Shanghai, China) following the manufacturer’s instructions. The kit sensitivities were <1 pg/mL for IL-2, 1 ng/L for IL-10, and 3 pg/mL for IFN-γ, according to the manufacturer’s specifications.

#### 2.4.3. Determination of the Splenic CD4^+^/CD8^+^ Ratio

Spleens were collected from chickens in each group when they were 21 and 28 days old. At each time point, three chickens were randomly selected from each subgroup. After removing the capsule, the spleens were cut into small pieces and gently mashed through a 70 μm cell strainer using a syringe plunger, with frequent rinsing using PBS to obtain single-cell suspensions [40]. Erythrocytes were removed via density gradient centrifugation using chicken spleen lymphocyte separation medium (Solarbio, Beijing, China). After erythrocyte depletion, 1 × 10^6^ cells were aliquoted into centrifuge tubes and resuspended in 100 μL of PBS. Each tube was stained with 7 μL of anti-chicken CD3 SPRD (clone CT-3, 0.1 mg/mL), anti-chicken CD4 FITC (clone CT-4, 0.5 mg/mL), and anti-chicken CD8 PE (clone EP72, 0.1 mg/mL) antibodies (SouthernBiotech, Birmingham, AL, USA), followed by incubation at room temperature in the dark for 30 min. After three washes with PBS, the cells were resuspended in 500 μL of flow cytometry staining buffer and analyzed for CD4^+^/CD8^+^ ratios by conducting flow cytometry (BD FACSCanto II, BD Biosciences, Franklin Lakes, NJ, USA). CD3-positive cells were first gated, and within this population, the percentages of CD4+ and CD8+ T cells were then determined.

#### 2.4.4. Anticoccidial Index (ACI)

The protective efficacy was evaluated using the ACI [41], which was calculated as follows: ACI = (survival rate + relative rate of weight gain) − (lesion score + oocyst value). Survival rate was determined as the percentage of chickens surviving until the end of the experiment in each group. Relative rate of weight gain was calculated using the formula: (weight gain rate of experimental group/weight gain rate of control group) × 100. Lesion score was assessed according to the method of Johnson and Reid [42] by personnel blinded to the group assignments based on the severity of intestinal lesions. Oocyst counts were determined using a McMaster counting chamber following standard protocols. This comprehensive index integrates multiple parameters to quantitatively assess vaccine efficacy against coccidial infection.

### 2.5. Safety Evaluation

The safety evaluation design addressed critical requirements for vaccines [43]. Multiple dosage groups (100 μg single dose, 100 μg booster, 300 μg high dose) were included to simulate clinical scenarios: standard immunization, repeated administration, and accidental overdosing. Hematological parameters and serum biochemistry were selected to detect potential toxicity to hematopoietic, hepatic, and renal systems—organs particularly vulnerable to vaccine-related adverse effects [44]. Long-term histopathological examination (15, 30, 60 days post-immunization) of major organs was critical, as food-producing animals require vaccines to be free of delayed toxicity, ensuring meat safety [45]. To assess the safety profile of the TEIN subunit vaccine, 100 one-day-old chickens were randomly allocated into four groups: a control group (Control) and three experimental groups (Groups A, B, and C). The control group received no treatment. In the experimental groups, chickens in Group A were intramuscularly injected with 100 μg of the TEIN subunit vaccine in the leg muscle at one day of age. Chickens in Group B received three intramuscular injections of 100 μg of the TEIN subunit vaccine at 7, 14, and 21 days of age. Chickens in Group C received a single intramuscular injection of 300 μg of the TEIN subunit vaccine at 14 days of age. All injections were administered at a volume of 100 μL per chicken. Throughout the experiment, all chickens were observed daily for mortality, general clinical status, and local injection site reactions. The detailed immunization schedule and dosage regimen are presented in Table 2. The safety evaluation included hematological analysis (complete blood count), serum biochemical profiling, and histopathological examination of major organs with hematoxylin and eosin (H&E) staining.

#### Hematological, Serum Biochemical, and Histopathological Analyses

For safety evaluation, whole blood samples were collected from the wing vein of chickens in each group at 7, 14, and 21 days after the final immunization using sterile syringes [38]. At each time point, 3 chickens were randomly selected from each group. From each chicken, blood samples were collected using both EDTA-2Na anticoagulant tubes (for hematological analysis) and tubes without anticoagulant (for serum biochemical analysis). Hematological parameters, including red blood cell count, white blood cell count, hemoglobin concentration, and hematocrit levels, were measured by Saivel Biological Co., Ltd. (Changchun, China). For serum biochemical analysis, all blood samples were processed immediately after collection to minimize nutrient consumption by nucleated erythrocytes. Serum was obtained via centrifugation at 3000 rpm for 15 min at 4 °C and analyzed by Saivel Biological Co., Ltd. (Changchun, China) via standard automated biochemistry assays. The following parameters were quantified: glucose, triglycerides, urea, total cholesterol, total protein, alanine aminotransferase, aspartate aminotransferase, and amylase.

For histopathological examination, at 15, 30, and 60 days post-final immunization, cardiac, hepatic, splenic, pulmonary, renal, and intestinal tissues were collected from each group. At each time point, 3 randomly selected chickens per group were euthanized for tissue collection. Tissues were fixed in paraformaldehyde for 24 h, then dehydrated, embedded in paraffin, and cut into sections (3 μm thick). After deparaffinization and alcohol gradient rehydration, the sections were stained with H&E for microscopic evaluation of pathological changes in major organs.

### 2.6. Statistical Analysis

All statistical analyses were performed using GraphPad Prism 8.0. The data are presented as the mean ± SD. Normality of the data was assessed using the Shapiro-Wilk test. For pairwise comparisons between two groups, a paired *t*-test was used. For comparisons involving multiple groups and time points, a two-way ANOVA was conducted followed by Šidák’s multiple comparisons test. Differences were considered statistically significant at *p* < 0.05.

## 3. Results

### 3.1. Preparation of TEIN Recombinant Protein

**Figure 1:**
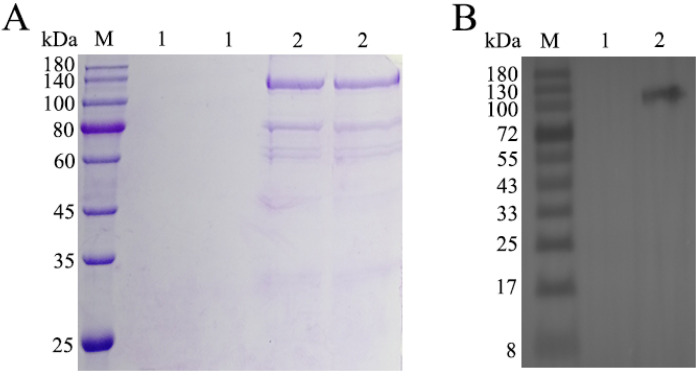
Purification of recombinant TEIN protein. (**A**) Sodium Dodecyl Sulfate–Polyacrylamide Gel Electrophoresis (SDS-PAGE) analysis of purified recombinant TEIN protein. The gel was stained with Coomassie Brilliant Blue. (**B**) Western blotting of the recombinant TEIN protein probed with an anti-His tag primary antibody. Lane M, prestained protein marker; Lane 1, pPIC9K empty vector; Lane 2, purified recombinant TEIN protein.

The purified TEIN protein was analyzed via SDS-PAGE, with pPIC9K serving as the control. The results of Coomassie Brilliant Blue staining and destaining are presented in Figure 1A. The predicted molecular weight of TEIN was 110 kDa, and it was observed at the expected molecular weight based on SDS-PAGE. The yield of the purified TEIN protein was approximately 20 mg/L of culture supernatant. Western blotting analysis of the TEIN protein (Figure 1B) revealed a specific band at 110 kDa, which corresponded well with the SDS-PAGE results.

### 3.2. Evaluation of the Immunoprotective Efficacy of the TEIN Subunit Vaccine

#### 3.2.1. Serum Anti-TEIN IgY Antibody Detection

**Figure 2:**
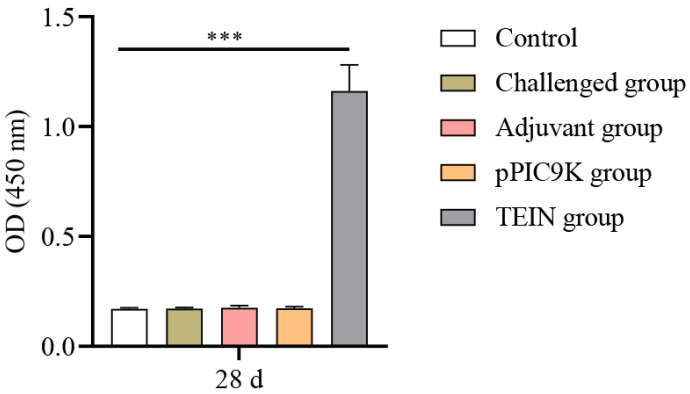
Changes in Immunoglobulin Y (IgY) levels in the serum of chickens in each group. Serum IgY concentrations were determined based on Enzyme-Linked Immunosorbent Assay (ELISA) at 28 days of age (OD 450 nm). Data are presented as mean ± SD (*n* = 5). Data were analyzed using an unpaired *t*-test. *** *p* < 0.001.

Serum levels of TEIN-specific IgY antibodies were quantified by conducting indirect ELISA at 28 days of age (Figure 2). Compared to the control group, the TEIN group presented significantly higher anti-TEIN IgY antibody levels (*p* < 0.001) at this time point. These results indicated that the TEIN subunit vaccine has excellent immunogenicity and effectively induces TEIN-specific IgY antibody production in chickens.

#### 3.2.2. The TEIN Subunit Vaccine Enhances Serum IL-2, IFN-γ, and IL-10 Levels in Chickens

**Figure 3:**
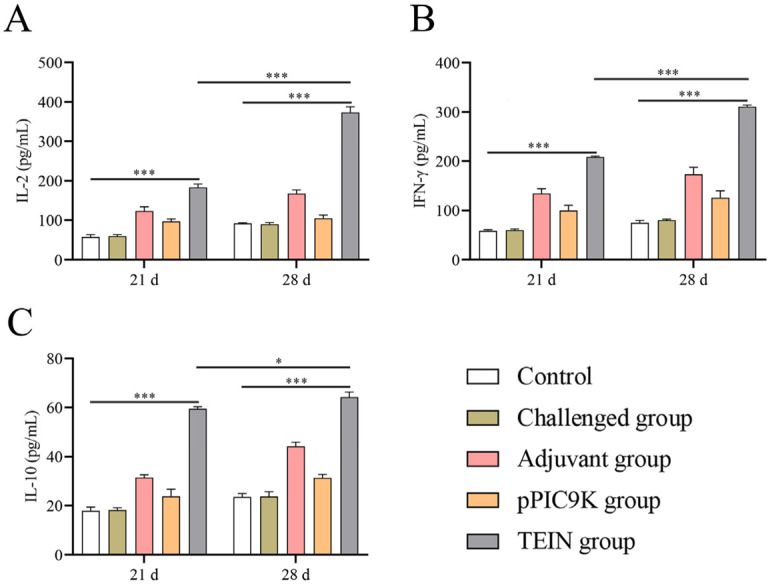
The levels of Interleukin-2 (IL-2), Interferon-γ (IFN-γ), and Interleukin-10 (IL-10) in the serum of chickens. The contents of IL-2 (**A**), IFN-γ (**B**), and IL-10 (**C**) in the serum of chickens in each group were detected using ELISA kits. Data are presented as mean ± SD (*n* = 3). Data were analyzed based on two-way ANOVA followed by Šidák’s multiple comparisons test. * *p* < 0.05 and *** *p* < 0.001.

Serum concentrations of IL-2, IFN-γ, and IL-10 were determined at 21 and 28 days of age in each group of chickens using commercial ELISA kits. Compared to the control group, the TEIN group presented significantly higher levels of IL-2 (Figure 3A), IFN-γ (Figure 3B), and IL-10 (Figure 3C) following primary immunization (*p* < 0.001). On day 28, the TEIN group presented significantly elevated levels of IL-2 and IFN-γ compared to the 21-day group (*p* < 0.001), whereas the IL-10 concentration exhibited a modest but significant increase (*p* < 0.05) (Figure 3). These results indicate that the TEIN vaccine effectively stimulated the production of IL-2, IL-10, and IFN-γ following primary immunization, with booster immunization resulting in a more pronounced increase in IL-2 and IFN-γ levels than the increase in IL-10 levels. These findings indicate that the TEIN subunit vaccine promotes a balanced immune response.

#### 3.2.3. The TEIN Subunit Vaccine Increases the Splenic CD4^+^/CD8^+^ Ratio in Chickens

**Figure 4:**
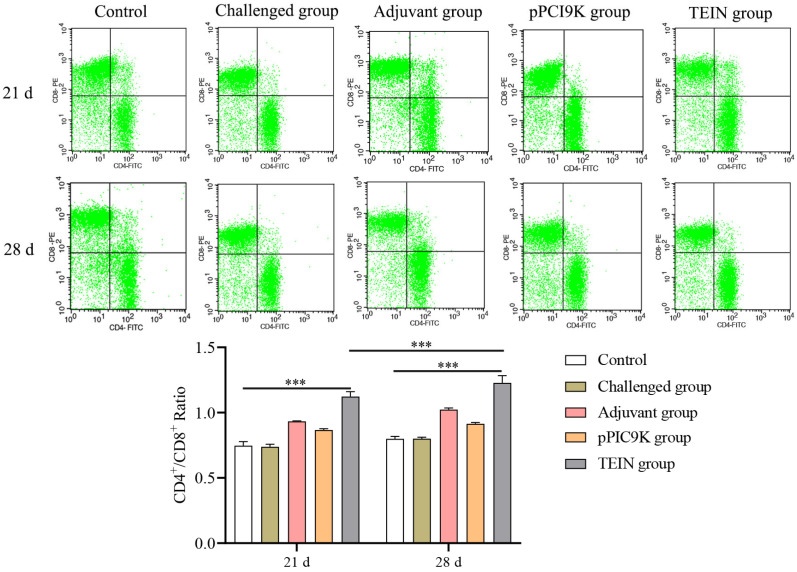
The CD4^+^/CD8^+^ ratio in the spleen was determined using flow cytometry. The CD4^+^/CD8^+^ ratios were determined using flow cytometry after staining with anti-chicken CD3 SPRD, anti-chicken CD4 FITC, and anti-chicken CD8 PE antibodies at the ages of 21 and 28 days. Data are presented as mean ± SD (*n* = 3). Data were analyzed using two-way ANOVA followed by Šidák’s multiple comparisons test. *** *p* < 0.001.

Chicken spleens from each group were collected at 21 and 28 days of age, made into single-cell suspensions after erythrocytes were removed, and then stained with anti-chicken CD3 SPRD, anti-chicken CD4 FITC, and anti-chicken CD8 PE flow antibodies to detect changes in the CD4^+^/CD8^+^ ratio. The TEIN group had significantly greater CD4^+^/CD8^+^ ratios at 21 and 28 days of age than the control group (*p* < 0.001) (Figure 4). Compared to that in the initial immunization group, the ratio of CD4^+^/CD8^+^ T cells was significantly greater in the TEIN group after booster immunization (*p* < 0.001). These results suggest that the TEIN subunit vaccine can enhance the immune function of chickens and improve the level of immune response in the body. An elevated CD4+/CD8+ ratio reflects enhanced T cell-mediated immunity, which plays a key role in protecting against intracellular parasites such as *Eimeria*. Therefore, the increased CD4+/CD8+ ratio observed in the TEIN group suggests that the vaccine may enhance protective immunity against coccidiosis.

#### 3.2.4. Anticoccidial Index Analysis

To evaluate the protective efficacy of the TEIN subunit vaccine against single infections with *E. tenella*, *E. acervulina*, *E. maxima*, and *E. necatrix* as well as mixed infections, the protective effect was comprehensively assessed using ACI (Table 3). Following immunization with the TEIN vaccine and subsequent challenge with each *Eimeria* species, the lesion scores were 14 for *E. tenella*, 10 for *E. acervulina*, 10 for *E. maxima*, 10.04 for *E. necatrix*, and 10 for mixed infection, whereas the lesion scores in the corresponding challenged groups ranged from 36 to 38. According to established criteria, ACI values ≥ 180 indicate good protection, values between 160 and 179 indicate moderate protection, and values below 160 indicate poor protection. The ACIs for the TEIN subunit vaccines against *E. tenella*, *E. acervulina*, *E. maxima*, and *E. necatrix*, single infections and mixed infections with all four species were 174.82, 174.58, 174.41, 180.61, and 175.95, respectively. These results demonstrate that the TEIN subunit vaccine provides effective protection against both single and mixed infections with these four *Eimeria* species.

### 3.3. Safety Evaluation of the TEIN Subunit Vaccine

Throughout the experimental period, no mortality or abnormal clinical signs were observed in any of the vaccinated groups. All chickens remained healthy with normal mental status, appetite, feather condition, and behavior, and no local injection site reactions were noted at any time point post-immunization.

#### 3.3.1. Hematological Parameters in Chickens After TEIN Vaccination

Complete blood counts were performed on samples collected at 7, 14, and 21 days post-final immunization, and the red blood cell count, white blood cell count, hemoglobin, and hematocrit were measured (Table S1). No significant differences were found between experimental groups (A, B, and C) and the control group (*p* > 0.05), indicating that administering the TEIN subunit vaccine at different doses and frequencies did not significantly affect hematological parameters. These findings demonstrate the absence of hematotoxicity or adverse effects associated with TEIN vaccination.

#### 3.3.2. Serum Biochemical Parameters in Chickens After TEIN Vaccination

Serum biochemical profiles were analyzed at 7, 14, and 21 days post-final immunization. No significant differences were observed between the experimental groups (A, B, and C) and the control group in the levels of glucose, triglycerides, urea, total cholesterol, total protein, alanine aminotransferase, aspartate aminotransferase, or amylase at any time point (*p* > 0.05; Table S2), confirming that TEIN vaccination did not adversely affect hepatic, renal, or metabolic functions. These results confirmed the safety profile of the TEIN subunit vaccine.

#### 3.3.3. Histopathological Evaluation of the Major Organs After TEIN Vaccination

**Figure 5:**
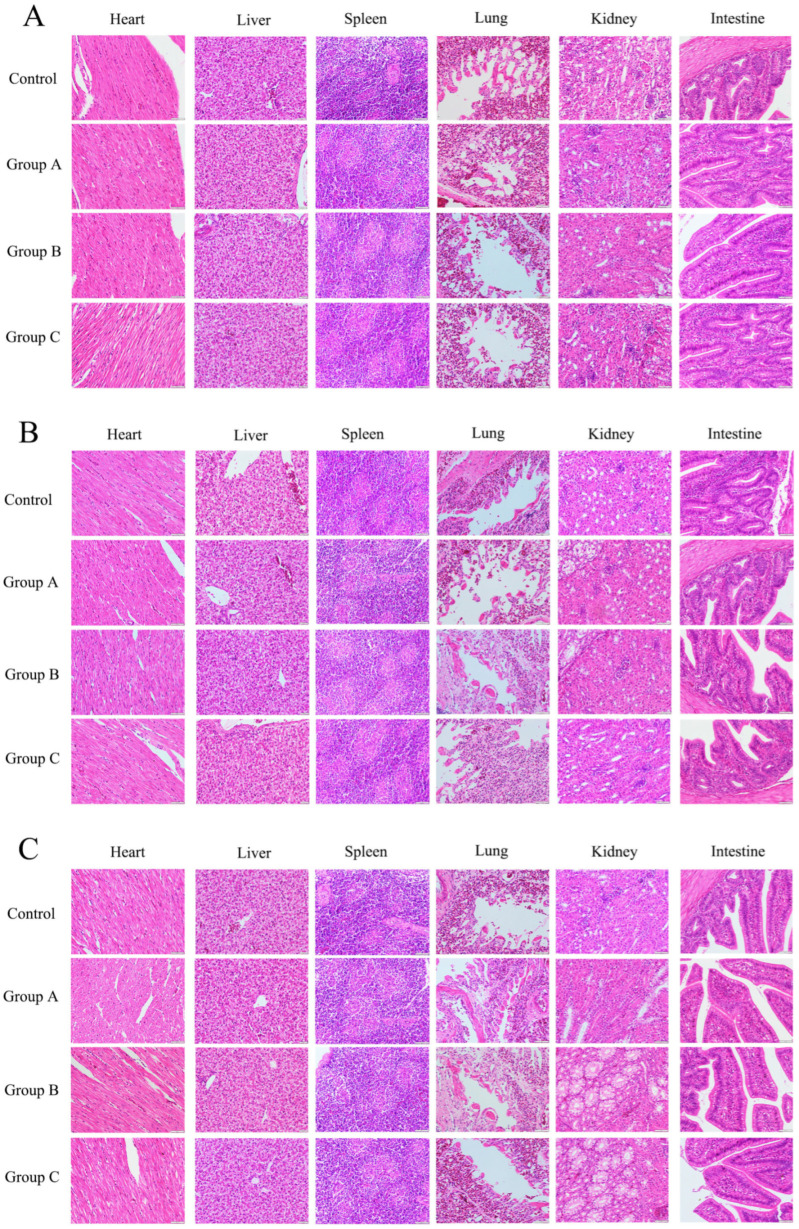
Histopathological analysis of major organs in chickens. Paraffin-embedded sections (3 μm) of major organs were prepared and examined for pathological changes by conducting Hematoxylin and Eosin (H&E) staining (original magnification: 400×). (**A**) 15 days post-final immunization, (**B**) 30 days post-final immunization, (**C**) 60 days post-final immunization.

Histopathological evaluation of cardiac, hepatic, splenic, pulmonary, renal, and intestinal tissues was conducted at 15, 30, and 60 days after the final immunization. In the event of vaccine-induced toxicity, expected pathological changes may include inflammatory cell infiltration, cellular necrosis, tissue degeneration, congestion, hemorrhage, or fibroplasia in target organs. However, microscopic examination revealed no significant pathological alterations in any of the examined organs, with representative images shown in Figure 5, confirming the absence of vaccine-induced tissue damage. These results suggest that TEIN vaccination results in a favorable safety profile in terms of histopathological changes in major organs.

## 4. Discussion

Under field conditions, chicken coccidiosis typically presents as mixed infections with multiple *Eimeria* species [46]. Current research indicates that an ideal coccidiosis vaccine should not only provide cross-protection against multiple *Eimeria* infections but also demonstrate high efficacy, safety, and production feasibility to meet practical farming requirements [47,48]. Notably, the *P. pastoris* expression system used in this study is well-established for industrial-scale fermentation, with reported yields of recombinant proteins reaching up to several grams per liter under optimized high-cell-density fermentation conditions [49]. The TEIN protein yield obtained in this study (20 mg/L from shake flask culture) represents the expression level without further fermentation optimization. Further optimization of fermentation processes could potentially increase the yield to levels suitable for commercial application [50].

Protective antigenic genes of *Eimeria* constitute the foundation for developing effective vaccines [51]. These genes primarily include those expressed during the sporozoite, merozoite, and gametocyte developmental stages [52,53,54]. Among these genes, the A4 gene is designated TA4 in *E. tenella* (known as SAG1) and NA4 in *E. necatrix*, both of which are predominantly expressed during the sporozoite stage and are involved in sporozoite adhesion and invasion. Vaccines targeting TA4 and NA4 have demonstrated protective efficacy against *E. tenella* and *E. necatrix* infections [34,55]. The 3-1E gene, which is expressed mainly during the sporozoite and schizont stages, encodes highly immunogenic proteins. Studies have shown that a 3-1E-based DNA vaccine reduces oocyst shedding in *E. acervulina*-infected chickens, achieving an ACI of 169.55 [56]. IMP1 is a highly conserved membrane protein in *E. maxima* [57,58]. Nanoparticle vaccines incorporating IMP1 have shown protective effects against *E. maxima* infection, improving feed conversion efficiency and body weight gain in challenged birds [59]. Subsequent studies confirmed that transgenic *Eimeria* vaccines expressing EmIMP1 significantly reduced intestinal lesions and inhibited the proliferation of *E. maxima* in commercial broilers [60].

Several recent studies have developed recombinant subunit vaccines against *Eimeria* infections, but most have focused on single-species protection [61,62,63,64], which limits their applicability in the field where mixed infections predominate. Although one study evaluated a single-antigen vaccine against mixed infections and reported reduced oocyst shedding [65], protection conferred by a single antigen may be insufficient to provide adequate protection against mixed infections with multiple *Eimeria* species. Unlike previous studies that focused on expressing a single *Eimeria* protein and only targeted single-species infections, in this study, the genes encoding *E. tenella* TA4, *E. maxima* 3-1E, *E. necatrix* IMP1, and *E. acervulina* NA4 were expressed in tandem to construct a TEIN subunit vaccine. The immunization schedule and dose were determined based on the protocol described by Huang [66]. Following immunization, chickens were challenged with 10,000 oocysts per chicken, a standardized dose widely adopted in previous studies within experimental challenge models [67,68], and the protective efficacy of the TEIN subunit vaccine against single infections of *E. tenella*, *E. necatrix*, *E. acervulina*, and *E. maxima* was evaluated by calculating the ACI. The results indicated that the TEIN subunit vaccine provided effective protection against *E. tenella*, *E. necatrix*, *E. acervulina*, and *E. maxima* infections in chickens, with ACIs of 174.82, 180.61, 174.58, and 174.41, respectively. These findings suggest that the tandem expression of multiple protective antigen genes from *Eimeria* species is a viable strategy to enhance the immunoprotective effects of vaccines against various coccidian parasites. Similarly, a multiepitope vaccine containing antigenic epitopes from *E. necatrix* NA4, *E. tenella* SAG1, *E. acervulina* LDH, and *E. maxima* CDPK was developed by Yu et al. [69] and showed high efficacy of protection against single infections of *E. necatrix*, *E. acervulina*, *E. tenella*, and *E. maxima*. The results revealed that chickens immunized with this multiepitope vaccine presented significantly better growth performance and a marked reduction in oocyst output in the intestinal contents when challenged with any of the four *Eimeria* species. However, the study did not assess the efficacy of the vaccine against mixed infections with these coccidian species. Unlike previous studies, the present one further evaluated the immunoprotective effect of the TEIN subunit vaccine on chickens with mixed infections of *E. tenella*, *E. necatrix*, *E. acervulina*, and *E. maxima* (the dominant clinical scenario). The results revealed that vaccination with the TEIN subunit vaccine significantly increased relative weight gain in chickens challenged with mixed infections, yielding an ACI of 175.95, while all groups maintained 100% survival throughout the experiment. This ACI value is similar to those obtained from single-species challenges, suggesting that the mixed infection model is suitable for evaluating vaccine efficacy under conditions mimicking natural exposure. These findings indicate that the TEIN subunit vaccine can provide effective immune protection against co-infections involving *E. tenella*, *E. necatrix*, *E. acervulina*, and *E. maxima*.

To evaluate the immunoprotective effects of TEIN vaccines, it is essential to assess IgY levels, CD4^+^/CD8^+^ ratios, and cytokine profiles, as these parameters reflect immune system activation and inflammatory modulation by TEIN subunit vaccines. IgY, an immunoglobulin abundantly present in birds, shares functional similarities with mammalian IgG and plays a protective role against *Eimeria* infections in avian species [70,71]. In this study, the constructed TEIN subunit vaccine significantly elevated serum IgY levels in chickens, with the TEIN group showing the highest IgY concentrations following the second immunization. These findings indicate that the TEIN subunit vaccine effectively activates humoral immunity, leading to the production of specific antibodies. CD4^+^ T cells function as helper T cells by secreting cytokines such as IL-2 and IFN-γ, which activate macrophages and B cells to mediate cellular and humoral immune responses, respectively, thereby increasing resistance to infection [72]. CD8^+^ T cells, as cytotoxic T cells, can directly eliminate coccidia-infected cells and suppress coccidia proliferation through IFN-γ secretion [73,74,75]. Coccidian infection in chickens significantly promotes the activation of CD4^+^ T cells and CD8^+^ T cells, which synergize to exert anti-infective effects and establish immune protection [76,77]. In this study, after chickens were immunized with the TEIN subunit vaccine, the CD4^+^/CD8^+^ ratios, along with the levels of IL-2 and IFN-γ, significantly increased, suggesting that the immune system of chickens was activated by the TEIN subunit vaccine. Additionally, IL-10, a crucial anti-inflammatory factor, can suppress excessive immune responses and maintain immune homeostasis [78,79]. The elevated IL-10 levels observed in this study suggest that the TEIN vaccine may prevent hyperactivation of the immune system through modulation of the inflammatory response. However, whether this immunoregulatory balance is associated with proper protein folding in *P. pastoris* requires further investigation.

With increasingly stringent requirements for disease prevention and control, vaccine safety has become important in both research and application [80]. In this study, the safety of the TEIN subunit vaccine was evaluated using hematological, serum biochemical, and histopathological analyses. Hematological parameters showed no significant differences between vaccinated and control groups, consistent with the approach used by Liu [81], who demonstrated DNA vaccine safety based on the absence of hematological abnormalities. Serum biochemical markers were selected to assess metabolic and organ health in poultry, as they provide reliable indicators of hepatic and renal function across different physiological stages [82]; no significant differences were observed between vaccinated and control groups, indicating that the TEIN vaccine did not adversely affect metabolic or organ function. Histopathological examination revealed no pathological alterations in major organs, supporting histopathology as a critical tool for vaccine safety monitoring, as emphasized by ref. [83]. Collectively, these complementary assessments demonstrate that the TEIN subunit vaccine exhibits a favorable safety profile in chickens.

Selection of an appropriate vaccine delivery route is critical for ensuring both efficacy and safety in poultry immunization. In this study, intramuscular injection into the leg muscle was selected as the delivery method for the TEIN subunit vaccine. This route has been widely employed in experimental poultry vaccine studies due to its ability to ensure consistent and accurate vaccine delivery, precise control of injection volume, and reliable induction of systemic immune responses [84]. Although individual intramuscular injection is labor-intensive and time-consuming, mass vaccination methods such as drinking water or spray (aerosol) administration often lack dose uniformity among individual birds during the delivery process, which may result in inadequate vaccine-induced immunity [85]. However, the use of intramuscular injection, particularly in day-old chicks, requires careful consideration of potential risks including muscle damage, injection site reactions, and handling stress. Indeed, alternative delivery routes have shown promise in poultry vaccination. For instance, oral administration of recombinant *Lactobacillus plantarum* expressing *E. tenella* antigens significantly increased intestinal IgA and serum IgG antibody titers, enhanced Th1 cytokine responses (IL-2 and IFN-γ), and reduced oocyst shedding following challenge infection [15]. Similarly, intranasal immunization with immunostimulatory complexes (ISCOMs) containing *E. tenella* antigens was demonstrated to be the most effective route for inducing protective immune responses compared to in ovo or intramuscular administration [86]. These findings suggest that while intramuscular injection remains a reliable method for experimental vaccine evaluation, the exploration of alternative administration routes could offer practical advantages for large-scale poultry immunization.

While the TEIN vaccine conferred effective protection against both single and mixed *Eimeria* infections under controlled laboratory conditions, its efficacy under complex field settings remains to be determined. In addition, the experimental period was relatively short, and long-term immunity as well as the duration of protection induced by the vaccine were not assessed. Furthermore, although key cytokines and T cell subsets were measured, the cellular immune mechanisms underlying the observed protection were not investigated in depth. The protective efficacy of the vaccine was also evaluated only in broilers, and its effect in laying hens remains unknown. Future studies, including field trials, long-term monitoring, and more detailed immunological analyses, are warranted to further validate the potential application of the TEIN vaccine.

## 5. Conclusions

In this study, the TEIN subunit vaccine was developed and its protective efficacy was evaluated against single and mixed infections with *E. tenella*, *E. necatrix*, *E. acervulina*, and *E. maxima* under controlled laboratory conditions. The vaccine exhibited a favorable safety profile and conferred protection against both single and mixed *Eimeria* infections in chickens. Further studies, including field trials to assess efficacy under natural farming conditions and evaluation of long-term protection, are warranted to support its potential application.

## Figures and Tables

**Table 1:** Vaccination strategies and challenge strategies.

Group	Vaccination Strategy		Challenge Strategy	
	Treatment (per Chicken)	Vaccination Time	Treatment (per Chicken)	Challenge Time
Control	—	—	—	—
Challenged group	—	—	1 × 10^4^ sporulated oocysts (*E. tenella* or *E. acervulina* or *E. maxima* or *E. necatrix* or mixed)	At 28 days of age
Adjuvant group	Aluminum hydroxide adjuvant	The first immunization and the second immunization were performed at 14 and 21 days of age		
pPIC9K group	100 μg pPIC9K empty protein			
TEIN group	100 μg TEIN Subunit vaccine			

**Table 2:** Experimental grouping and immunization schedule.

Age Groups	Day 1	Day 7	Day 14	Day 21
Control	—	—	—	—
Group A	100 μg TEIN	—	—	—
Group B	—	100 μg TEIN	100 μg TEIN	100 μg TEIN
Group C	—	—	300 μg TEIN	—

**Table 3:** Protective efficacy of the TEIN subunit vaccine against different species of *Eimeria* coccidia-infected chickens.

Groups	Challenge with *Eimeria* spp.	Survival Rate (%)	Relative Weight Gain Rate (%)	Oocyst Value	Lesion Score Value	ACI
Control	*E. tenella*	100	100	0	0	200
Challenged group		100	40.94	20	36	84.94
Adjuvant group		100	42.36	20	32	90.36
pPIC9K group		100	41.22	20	38	83.22
TEIN group		100	89.82	1	14	174.82
Control	*E. acervulina*	100	100	0	0	200
Challenged group		100	37.38	10	38	89.38
Adjuvant group		100	45.68	10	34	101.68
pPIC9K group		100	40.28	10	34	96.28
TEIN group		100	85.58	1	10	174.58
Control	*E. maxima*	100	100	0	0	200
Challenged group		100	38.35	10	38	90.35
Adjuvant group		100	40.24	10	36	94.24
pPIC9K group		100	39.46	10	38	91.46
TEIN group		100	85.41	1	10	174.41
Control	*E. necatrix*	100	100	0	0	200
Challenged group		100	41.24	10	36	95.24
Adjuvant group		100	45.88	10	38	97.88
pPIC9K group		100	41.68	10	38	93.68
TEIN group		100	91.65	1	10.04	180.61
Control	Mixed	100	100	0	0	200
Challenged group		100	38.09	10	38	90.09
Adjuvant group		100	39.86	10	38	91.86
pPIC9K group		100	36.26	10	38	88.26
TEIN group		100	86.95	1	10	175.95

## Data Availability

The raw data supporting the conclusions of this article will be made available by the authors upon request.

## Supplementary Materials

The following supporting information can be downloaded at: https://www.mdpi.com/article/10.3390/ani16071087/s1, Figure S1: Schematic diagram of the recombinant plasmid pPIC9K-TEIN; Table S1: Hematological parameters in chickens following TEIN subunit vaccination; Table S2: Serum biochemical parameters in chickens following TEIN subunit vaccination.

## Abbreviations

The following abbreviations are used in this manuscript:

*P. pastoris*	*Pichia pastoris*
IgY	Immunoglobulin Y
IL-2	Interleukin-2
IL-10	Interleukin-10
IFN-γ	Interferon-gamma
ACI	Anticoccidial Index
SDS-PAGE	Sodium dodecyl sulfate–polyacrylamide gel electrophoresis
HRP	Horseradish peroxidase
PBST	Phosphate-buffered saline with Tween-20
BCA	Bicinchoninic acid
MD plates	Histidine-deficient minimal dextrose plates
YPD medium	Yeast extract-peptone-dextrose medium
BMGY medium	Buffered glycerol-complex medium
BMMY medium	Buffered methanol-complex medium
Ni-NTA	Nickel-nitrilotriacetic acid
H&E	Hematoxylin and eosin stain
