# Supplementary material for: A Tetravalent Recombinant Subunit Vaccine Provides Protection Against Mixed Challenges with Four Eimeria Species in Chickens
Source: Animals (Basel). 2026 Apr 1;16(7):1087. doi: 10.3390/ani16071087 (PMC13072015; doi:10.3390/ani16071087)
Supplement: Supplementary file 1 [file animals-16-01087-s001.zip › Supplementary Tables S1-S2.pdf]

Table S1. Hematological parameters in chickens following TEIN subunit vaccination.

Test Item	Days Post Last Immunization	Control	Group A	Control	Group B	Control	Group C
Red Blood Cell count ($\times 10^{12}$/L)	7	2.72 ±0.03	2.70 ±0.06	2.52 ±0.11	2.62 ±0.10	2.83 ±0.07	2.82 ±0.09
	14	2.67 ±0.05	2.81 ±0.02	2.59 ±0.09	2.76 ±0.06	2.61 ±0.08	2.60 ±0.04
	21	2.59 ±0.10	2.75 ±0.03	2.61 ±0.21	2.72 ±0.13	2.90 ±0.40	2.81 ±0.27
White Blood Cell count ($\times 10^{9}$/L)	7	106.62 ±0.49	105.97 ±0.34	104.92 ±0.83	106.02 ±1.03	106.04 ±0.93	105.79 ±0.98
	14	106.08 ±0.69	105.78 ±0.591	2.52 ±0.11	106.48 ±1.10	105.38 ±0.72	106.07 ±1.03
	21	105.98 ±0.69	105.64 ±0.60	105.03 ±1.04	105.95 ±0.98	105.94 ±0.97	106.03 ±1.08
Hemoglobin (g/L)	7	134.67 ±2.51	131.39 ±2.65	106.13 ±1.02	134.97 ±1.93	135.07 ±1.93	134.97 ±2.05
	14	136.47 ±1.49	135.78 ±1.94	135.29 ±1.05	136.01 ±1.39	135.98 ±1.82	135.89 ±1.91
	21	135.92 ±2.48	136.03 ±2.04	134.96 ±1.20	136.76 ±1.89	136.40 ±1.79	135.87 ±1.68
Hematocrit (%)	7	32.97 ±1.03	32.50 ±0.94	136.21 ±1.97	32.97 ±0.69	33.07 ±0.98	32.94 ±1.24
	14	33.73 ±1.29	34.20 ±0.68	33.72 ±1.39	34.91 ±0.68	33.73 ±1.29	34.28 ±0.87
	21	34.02 ±1.56	34.16 ±1.01	33.93 ±1.40	33.98 ±0.97	33.91 ±1.01	34.01 ±1.07

Table S2. Serum biochemical parameters in chickens following TEIN subunit vaccination.

Test Item	Days Post Last Immunization	Control	Group A	Control	Group B	Control	Group C
Glucose (mmol/L)	7	13.39 ±1.37	12.63 ±0.93	13.94 ±0.97	13.03 ±0.93	13.39 ±1.37	12.63 ±0.93
	14	18.43 ±0.39	18.93 ±1.12	19.13 ±0.69	18.98 ±0.98	18.43 ±0.39	18.93 ±1.12
	21	19.67 ±1.01	20.03 ±0.96	17.97 ±0.87	21.07 ±0.65	19.00 ±1.32	18.68 ±1.56
Triglycerides (mmol/L)	7	0.59 ±0.26	0.63 ±0.28	0.69 ±0.28	0.70 ±0.29	0.59 ±0.26	0.63 ±0.28
	14	0.94 ±0.37	1.01 ±0.10	1.04 ±0.57	1.23 ±0.30	0.94 ±0.37	1.01 ±0.10
	21	1.03 ±0.42	1.26 ±0.47	1.24 ±0.67	1.46 ±0.24	0.69 ±0.65	0.95 ±0.69
Urea (mmol/L)	7	0.62 ±0.16	0.87 ±0.10	0.72 ±0.20	0.77 ±0.28	0.62 ±0.16	0.87 ±0.10
	14	0.79 ±0.12	0.89 ±0.11	0.99 ±0.35	1.14 ±0.31	0 .79 ±0.12	1.09 ±0.11
	21	0.80 ±0.28	0.92 ±0.16	1.04 ±0.68	1.24 ±0.46	0 .87 ±0.16	0.97 ±0.59
Total Cholesterol (mmol/L)	7	2.69 ±0.16	2.87 ±0.09	2.89 ±0.36	3.07 ±0.19	2.69 ±0.16	2.87 ±0.09
	14	2.79 ±0.13	2.91 ±0.11	2.89 ±0.32	2.79 ±0.40	2.79 ±0.13	2.91 ±0.11
	21	2.86 ±0.54	2.88 ±0.23	2.85 ±0.36	3.09 ±0.46	2.93 ±0.57	2.86 ±0.54
Total Protein (g/L)	7	19.39 ±1.57	19.63 ±1.93	20.39 ±1.07	19.70 ±0.93	19.39 ±1.57	19.63 ±1.93
	14	18.93 ±1.39	18.83 ±1.03	19.93 ±1.19	18.98 ±1.13	18.93 ±1.39	18.53 ±1.03
	21	19.45 ±1.60	20.02 ±1.59	20.67 ±1.47	20.83 ±0.99	18.57 ±1.35	18.38 ±0.99
Alanine Aminotransferase (U/L)	7	17.89 ±1.21	18.93 ±2.03	15.09 ±1.01	18.93 ±1.93	17.89 ±1.21	18.93 ±2.03
	14	18.10 ±2.09	19.03 ±1.83	20.80 ±1.99	20.93 ±1.03	18.10 ±2.09	19.03 ±1.83
	21	19.94 ±1.95	18.88 ±1.45	23.92 ±1.44	21.35 ±1.24	17.99 ±3.00	19.65 ±2.43
Aspartate Aminotransferase (U/L)	7	259.39 ±11.57	259.04 ±19.93	249.39 ±13.57	260.04 ±18.93	259.39 ±11.57	259.04 ±19.93
	14	262.93 ±21.39	261.93 ±20.03	249.33 ±19.39	259.09 ±20.13	262.93 ±21.39	258.93 ±20.03
	21	261.35 ±17.42	260.87 ±21.12	251.35 ±13.25	260.14 ±19.35	261.65 ±18.99	259.43 ±23.06
Amylase (U/L)	7	299.39 ±13.57	299.04 ±39.93	290.59 ±23.57	289.04 ±38.93	289.39 ±13.57	299.04 ±39.93
	14	332.93 ±31.39	331.93 ±30.93	340.93 ±29.39	318.59 ±33.59	332.93 ±31.39	328.93 ±30.93
	21	318.78 ±20.43	318.34 ±27.32	337.21 ±27.04	323.78 ±32.56	320.48 ±26.59	330.21 ±32.68
