# Supplementary figures and images for: A Tetravalent Recombinant Subunit Vaccine Provides Protection Against Mixed Challenges with Four Eimeria Species in Chickens
Source: Animals (Basel). 2026 Apr 1;16(7):1087. doi: 10.3390/ani16071087 (PMC13072015; doi:10.3390/ani16071087)

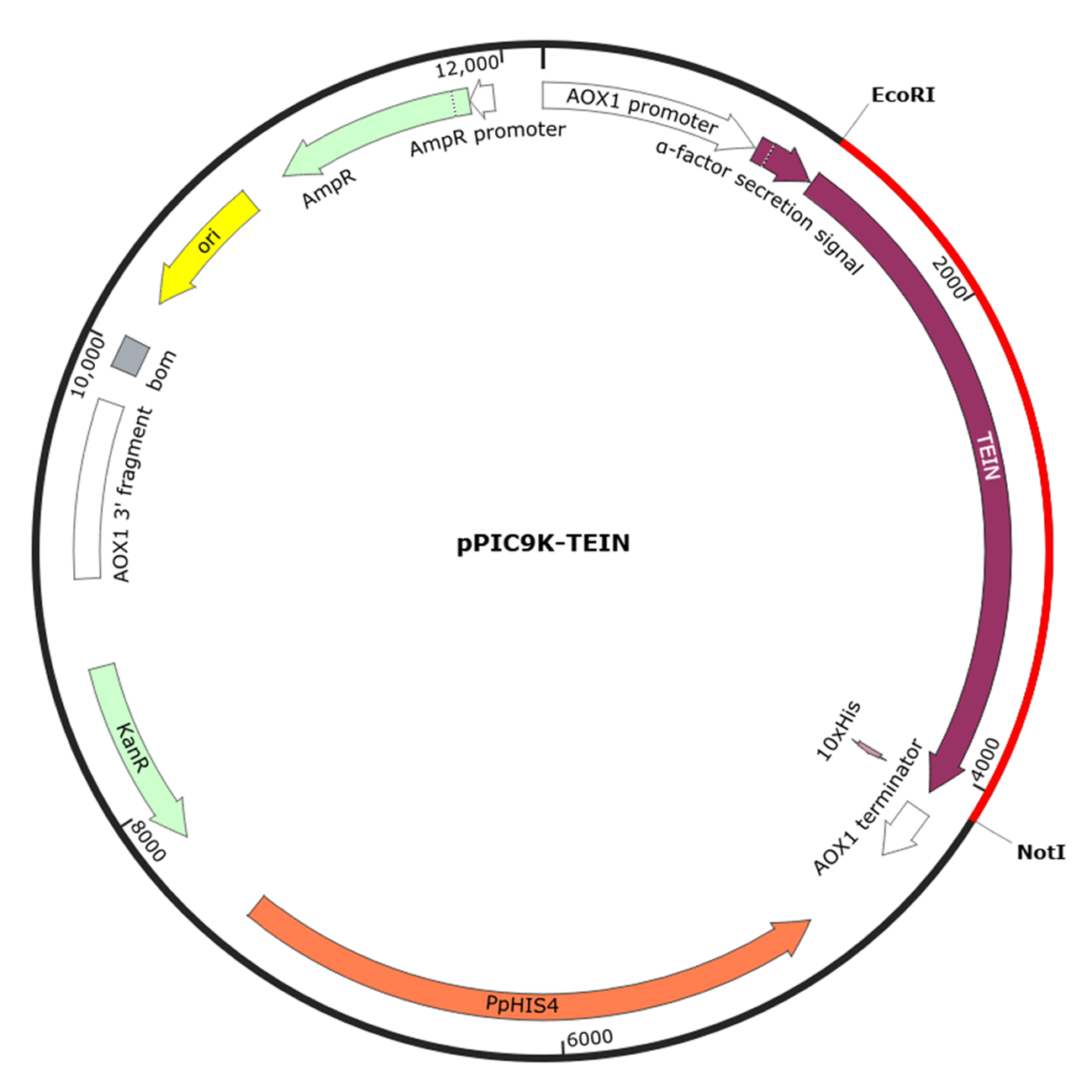

Supplement: Supplementary file 1 [file animals-16-01087-s001.zip › Figure S1. Schematic diagram of the recombinant plasmid pPIC9K-TEIN.png]
